# Cortical Cerebral Microinfarcts in Spontaneous Intracerebral Hemorrhage on 3T and 7T MRI

**DOI:** 10.1212/WNL.0000000000213642

**Published:** 2025-06-19

**Authors:** Wilmar M.T. Jolink, Susanne J. van Veluw, Doeschka A. Ferro, Kim Wiegertjes, Ingeborg Rasing, Jeroen Hendrikse, Marianne A.A. van Walderveen, Gabriel J.E. Rinkel, Marieke J.H. Wermer, Floris H.B.M. Schreuder, Catharina J.M. Klijn

**Affiliations:** 1 Department of Neurology, Isala, Zwolle, the Netherlands;; 2 Department of Neurology, J. Philip Kistler Stroke Research Center, Massachusetts General Hospital and Harvard Medical School, Boston;; 3 Department of Neurology, Rijnstate Hospital, Arnhem, the Netherlands;; 4 Department of Neurology, Donders Institute for Brain, Cognition and Behaviour, Center for Neuroscience, Radboud University Medical Center, Nijmegen, the Netherlands;; 5 Department of Neurology, Leiden University Medical Center, the Netherlands;; 6 Department of Radiology, University Medical Center Utrecht, the Netherlands;; 7 Department of Radiology, Leiden University Medical Center, the Netherlands;; 8 Department of Neurology and Neurosurgery, University Medical Center Utrecht Brain Center, the Netherlands; and; 9 Department of Neurology, University Medical Center Groningen, the Netherlands.

## Abstract

**Background and Objectives:**

MRI markers of cerebral small vessel disease (cSVD) in patients with spontaneous intracerebral hemorrhage (sICH) have provided insight in the underlying vascular pathology. Cortical cerebral microinfarcts (CMIs) are a relatively novel marker of cSVD. We aimed to assess presence and location within the cortex of CMIs in patients with sICH on 3T and 7T MRI, and to determine their association with hematoma location and other neuroimaging markers of cSVD.

**Methods:**

From a multicenter prospective observational cohort study, we included consecutive adult patients who underwent 3T and/or 7T MRI within 3 months after sICH. We determined presence and location of CMIs within the cortex (superficial or deeper layers; on 7T MRI) and assessed associations between presence of cortical CMIs and vascular risk factors, hematoma location, and other MRI markers of cSVD with multiple regression analyses.

**Results:**

We included 135 patients (mean age 63 years; 30% female). On 3T MRIs, we found 100 cortical CMIs in 57 patients (42%, median number 2, interquartile range [IQR] 1–3; lobar ICH 24 of 58 patients [41%]; non-lobar ICH 33 of 77 patients [43%]). On 7T MRI images, we found 59 cortical CMIs in 28 of 40 patients (70%; median number of 2, IQR 1–3; lobar ICH 8 of 12 patients [67%]; non-lobar ICH 20 of 28 patients [71%]). Presence of cortical CMIs was associated with history of TIA/ischemic stroke (relative risk [RR] 2.7, 95% CI 1.1–6.4), but not with other vascular risk factors or any of the MRI markers of cSVD. On 7T MRI, CMIs were located in the superficial part of the cortex in 30% of patients with lobar vs 5% of patients with non-lobar ICH (RR 2.7, 95% CI 1.5–5.0).

**Discussion:**

Cortical CMIs are a common finding in patients with sICH on both 3T and 7T MRIs with a similar frequency in patients with lobar and non-lobar sICH. Patients with lobar sICH, more often have CMIs in the superficial part of the cortex than those with non-lobar sICH. Location of CMIs within the cortex might provide insight into the underlying type of small vessel disease in sICH.

## Introduction

Spontaneous intracerebral hemorrhage (sICH) is a devastating disease affecting more than 3 million people worldwide each year.^1,2^ However, its etiology remains poorly understood. Traditionally, lobar sICH in elderly patients has been associated with cerebral amyloid angiopathy (CAA), but other factors likely play a role too, because pathology (mostly postmortem) studies found CAA in only 57% of patients with lobar sICH.^3^ In another autopsy study, only 16% of the patients with lobar ICH had CAA alone, while 42% had CAA combined with arteriolosclerosis.^4^ Non-lobar sICH is most commonly related to hypertension, but other vascular risk factors have also been identified.^5^ In a recent systematic review and meta-analysis, we showed that diabetes and alcohol overuse increase the risk of non-lobar sICH, and that hypertension is an important risk factor for both lobar and non-lobar sICH. These findings suggest overlapping disease mechanisms in lobar and non-lobar sICH.^6^

MRI markers of cerebral small vessel disease (cSVD) have provided insight in the underlying vascular disease in sICH. Strictly lobar cerebral microbleeds (CMBs),^7^ cortical superficial siderosis (cSS),^8^ white matter hyperintensities (WMHs) in a subcortical multispot pattern,^9,10^ and a severe degree of visible perivascular spaces (PVSs) in the white matter centrum semiovale^11^ are indicative of CAA in patients with or without a lobar sICH.^12^ Deep CMBs,^7^ peri-basal ganglia WMH,^9^ and PVS in the basal ganglia^11^ are more commonly found in arteriolosclerotic vasculopathy and non-lobar sICH.^13^ In another study, we found that enhancement on postgadolinium fluid-attenuated inversion recovery (FLAIR) MRIs, as a marker of blood-CSF barrier disruption, may be an additional marker of the underlying cSVD because it may be related to CAA.^14^

Advances in ultra-high-field MRI have made it possible to visualize submillimeter lesions. This allows the detection of cortical cerebral microinfarcts (CMIs), small lesions of presumed ischemic origin. In a small case series, we previously found cortical CMIs on 7T MRI in 75% of patients with sICH (6/6 lobar and 3/6 deep sICH).^15^ In a postmortem study combining 7T ex vivo MRI and histopathology, we observed that cortical CMIs were more prevalent in 9 patients with lobar sICH than in 11 patients with non-lobar sICH.^16^ Of interest, an exploratory analysis revealed that CMIs were predominantly located in the superficial layers of the cortex in lobar sICH patients, whereas in non-lobar sICH patients, CMIs were more often located in the deeper layers of the cortex.^16^ This observation suggests that the location of CMIs within the cortex might be indicative of the underlying type of small vessel disease.

In this study, we aimed to further investigate CMIs as a biomarker of cSVD on 3T and 7T MRI in patients with sICH by determining their prevalence, location in the cortex, and associations with vascular risk factors; hematoma location; and other neuroimaging manifestations of cSVD.

## Methods

### Study Population

We included patients from the Finding the ETiology in spontaneous Cerebral Hemorrhage (FETCH) study, a multicenter prospective observational cohort study in the Netherlands. In FETCH, adult patients were included with sICH confirmed by CT, who presented to the University Medical Centers of Nijmegen, Leiden, or Utrecht between October 1, 2013, and December 31, 2018. We selected the patients who underwent 3T and/or 7T MRI within 3 months of the sICH. We excluded patients with a known cause of the ICH on CTA, such as a vascular malformation, and patients with other known causes such as a bleeding disorder or tumor.

### Standard Protocol Approvals, Registrations, and Patient Consents

The FETCH study was approved by the Medical Ethics Review Committee of the University Medical Center Utrecht. Each patient gave written informed consent for participation in the study, and we followed the guidelines according to the Declaration of Helsinki.

### Vascular Risk Factors

Demographics and vascular risk factors were recorded on admission. We defined history of hypertension as use of antihypertensive medication, or a documented systolic blood pressure higher than 140 mm Hg or a diastolic blood pressure higher than 90 mm Hg on 2 independent measurements before the sICH. A transient ischemic attack, ischemic stroke, intracerebral and subarachnoid hemorrhage, atrial fibrillation, peripheral artery disease, and cardiac disease were extracted from the medical history. Diabetes mellitus was defined as diabetes in medical history or 2 fasting glucose measurements above 7 mmol/L and hypercholesterolemia as total cholesterol above 6.2 mmol/L or use of lipid-lowering drugs. We defined smoking as current or past tobacco use. We registered use of alcohol and defined alcohol overuse as ≥15 units/week. Patients with possible or probable CAA were identified using the Boston 2.0 criteria.^12^

### Imaging Protocol and Analysis

The imaging protocol and analysis have been described in detail before.^14^ In summary, on admission CT, we assessed sICH location, subarachnoid and intraventricular extension, and hematoma volume in mL using an in-house developed tool.^17^ Lobar sICH was defined as sICH in the cortex with or without involvement of subcortical white matter and non-lobar sICH as deep (thalamus and basal ganglia) or infratentorial (brainstem and cerebellum) sICH.^18^ We classified the ICH subtype (CAA, arteriolosclerosis, or mixed small vessel disease) according to the Causal Classification System for Intracerebral Hemorrhage Subtypes (CLAS-ICH).^13^

3T MRI (Philips Healthcare, Best, the Netherlands, and Siemens Healthineers, Erlangen, Germany) scans were acquired by a standardized protocol including 3D T_1_-weighted images, diffusion-weighted images (DWIs) with apparent diffusion coefficient (ADC) map, an axial T_2_*, T_2_-proton density weighted sequence, inversion recovery and FLAIR, all with 48 contiguous slices and 0.96 × 0.95 × 3.00 mm^3^ voxels.^14,19,20^

7T MRI (Philips Healthcare, Best, the Netherlands) scans were acquired by a standardized protocol including 3D T_2_-weighted images, 3D T_1_-weighted images, dual echo susceptibility-weighted images (SWIs), and 3D FLAIR.^14^ We administered the gadolinium-containing contrast agent in a single intravenous injection of 0.1 mL Gadovist/kg body weight with a maximum of 10 mL Gadovist or 0.2 mL Dotarem/kg body weight with a maximum of 30 mL Dotarem. Postgadolinium FLAIR images were acquired at least 10 minutes after contrast injection.

CMIs on 3T MRI were annotated by 1 trained reader (W.M.T.J.) blinded for patients' information. A random sample of 20% was assessed independently by a second expert reader (S.J.v.V.). Because of many discrepancies, a third trained reader (D.A.F.) checked all CMIs annotated by both readers and made the final call. All CMIs and contrast leakage on 7T MRI were annotated by 2 trained readers (W.M.T.J. and S.J.v.V.) and discussed in a consensus meeting.

Other cSVD MRI markers (CMBs, WMHs, cSS, lacunes, PVS, and DWI lesions) were assessed on 3T MRI by 1 trained rater, blinded for patient information, with a second rater assessing a random sample of at least 10% independently as previously described.^20,21^ For all MRI markers, discrepancies were discussed in a consensus meeting and, if necessary, discussed with an experienced neuroradiologist (M.A.A.v.W., J.H.) and neurologist (C.J.M.K.).

CMIs were assessed on FLAIR and T_1_-weighted images and defined as hypointense on T_1_, visible signal alteration on FLAIR, strictly intracortical and ≤3 mm. They had to be distinct from the hematoma (not <1 cm or in the adjacent gyrus), PVS, CMBs and leptomeningeal vessels, and visible in at least 2 planes (sagittal, transversal, and coronal).^22^ On 7T MRI, we determined the location of the CMIs in the cortex as superficial (touching the outer border of the cortex, predominantly in the upper half of the cortex), deep (not touching the outer border of the cortex, predominantly in the lower half of the cortex), or transcortical (covering more than 50% of the cortex). We assessed presence and extent of contrast leakage on 7T MRI.^14^ Contrast leakage was defined as a hyperintense signal in normal appearing brain or CSF on delayed postgadolinium FLAIR images, while absent on precontrast images. The signal had to be visually distinct and anatomically noncontiguous with the hematoma. We used the 5-point hyperacute injury marker (HARM) rating scale to rate the extent of the contrast leakage.^14,23^ CMBs, cSS, incidental DWI-positive lesions, lacunes, and WMH were rated in accordance with the Standards for Reporting Vascular Changes on Neuroimaging 2 definitions and the Microbleed Anatomical Rating Scale.^24,25^ In addition, we assessed the WMH volume by segmenting the WMH in the ICH-unaffected hemisphere using intensity-based thresholding in MRIcro^26^ with manual adjustment by one of the 2 trained readers, and we subsequently doubled this value to approximate the WMH volume in the ICH-affected hemisphere. We also expressed the WMH volumes as a percentage of intracranial volume (ICV).^20^ Only DWI lesions remote (>5 mm) from the hematoma and the area of perihematomal edema were scored.^27^ PVS were rated in basal ganglia and white matter centrum semiovale regions on axial 3T MRI T_2_-weighted sequences using a validated rating scale.^28^ We determined presence and extent (focal or disseminated) of the cSS.^8^

### Statistical Analysis

We analyzed group differences in presence, frequency, and distribution of clinical characteristics; hematoma characteristics; CMBs (presence [yes/no] and number per location [lobar, non-lobar and total] of CMBs); WMH (median Fazekas score, volume [in milliliters], and volume as percentage of ICV); DWI lesions (yes/no); PVS in basal ganglia and centrum semiovale (number ≥20, yes/no); cSS (presence [yes/no] and extent [focal or disseminated]) and lacunes (presence [yes/no]) on 3T MRI and contrast leakage (presence [yes/no]); and a severe HARM scale score (≥3, yes/no) on 7T MRI between patients with and without CMIs using the independent sample *t* test, χ^2^ test, and Mann-Whitney U as appropriate. We used multivariable logistic regression analysis to adjust for age and sex and all statistically significant (*p* < 0.05) variables. We used the rule of thumb of a minimum 10–15 outcomes per covariate. We also tested differences in superficial cortical localization, deep cortical localization, and transcortical localization of CMIs on 7T MRI between lobar and non-lobar sICH and according to CLAS-ICH etiology (CAA and mixed SVD vs arteriolosclerosis) using χ^2^ tests.

### Data Availability

The data that support the findings of this study are available from the corresponding or senior authors on reasonable request.

## Results

### 3T MRI

One hundred fifty-five of the in total 204 patients enrolled in the FETCH study underwent 3T MRI. Of the 155 patients who underwent 3T MRI, 20 patients were excluded because of insufficient quality of the T_1_ and/or FLAIR images to assess CMIs, mainly due to movement artefacts. Patients who underwent 3T MRI were younger and more often had a lobar ICH than the 49 patients who did not.^19^ Hematoma volume and NIH Stroke Scale (NIHSS) score at presentation were lower in patients who did undergo 3T MRI, but this was not significantly different. Baseline characteristics of the 135 included patients are given in Table 1. Patients had a mean age of 63 years (SD 12 years), and 30% were women. The median interval between sICH and 3T MRI was 9 days (interquartile range [IQR] 5–43 days). Median hematoma volume was 14 mL (IQR 5–30 mL). Location of the sICH was lobar in 58 patients (43%) and non-lobar in 77 patients (57%; 53 deep, 24 infratentorial). Thirty-two of 58 patients (55%) with lobar sICH fulfilled the Boston criteria version 2.0 for probable CAA.^12^ Of these 32 patients with probable CAA, 15 patients also had a history of hypertension. The ICH subtype according to the CLAS-ICH was CAA in 39 patients (29%), arteriolosclerosis in 45 patients (33%), and mixed SVD in 50 patients (37%).

**Table 1:** Baseline Characteristics for Patients With Lobar and Non-Lobar sICH With and Without Presence of Cortical CMIs on 3T MRI

Characteristics	All (N = 135)	CMI+ (N = 57, 42%)	CMI− (N = 78, 58%)	Risk ratio (95% CI)	*p* Value
Patient characteristics					
Age, y, mean (SD)	63 (14)	66 (13)	61 (15)		0.04^a^
Female sex, n (%)	40 (30)	17 (30)	23 (30)	1.0 (0.7–1.4)^c^	
GCS at presentation, median (IQR)	15 (13–15)	15 (13–15)	15 (13–15)		0.69^b^
NIHSS at presentation, median (IQR)	6 (3–11)	6 (2–12)	6 (3–12)		0.54^b^
Systolic BP at admission, mm Hg, mean (SD)	169 (32)	171 (30)	168 (34)		0.65^a^
Diastolic BP at admission, mm Hg, mean (SD)	94 (20)	94 (19)	93 (21)		0.94^a^
History of hypertension, n (%)	71 (53)	33 (58)	38 (49)	1.2 (0.9–1.6)^c^	
History of TIA or ischemic stroke, n (%)	17 (13)	13 (23)	4 (5)	2.7 (1.1–6.4)^c^	
History of ICH, n (%)	3 (2)	1 (2)	2 (3)	0.9 (0.4–1.9)^c^	
History of SAH, n (%)	1 (1)	1 (2)	0 (0)		
History of cardiac disease, n (%)	9 (7)	3 (5)	6 (8)	0.9 (0.5–1.4)^c^	
History of peripheral artery disease, n (%)	7 (5)	2 (4)	5 (6)	0.8 (0.5–1.3)^c^	
Diabetes mellitus, n (%)	16 (12)	9 (16)	7 (9)	1.4 (0.8–2.4)^c^	
Hypercholesterolemia, n (%)	43 (32)	18 (32)	25 (32)	0.99 (0.7–1.3)^c^	
Atrial fibrillation, n (%)	18 (13)	6 (11)	12 (15)	0.8 (0.6–1.2)^c^	
Smoking (current or past), n (%)	78 (58)	32 (56)	46 (59)	0.96 (0.7–1.3)^c^	
Alcohol use, n (%)	89 (66)	37 (65)	52 (67)	1.0 (0.7–1.4)^c^	
Alcohol use ≥15 units/week, n (%)	16 (12)	9 (16)	7 (9)	1.4 (0.8–2.5)^c^	
Anticoagulants, n (%)	29 (22)	13 (23)	16 (21)	1.1 (0.7–1.5)^c^	
Antiplatelets, n (%)	28 (21)	12 (21)	16 (21)	1.0 (0.7–1.5)^c^	
Hematoma characteristics					
Location of hematoma, n (%)					
Lobar	58 (43)	24 (42)	34 (44)		0.86^c^
Deep	53 (39)	22 (39)	31 (40)		
Infratentorial	24 (18)	11 (19)	13 (17)		
Hematoma volume, mL, median (IQR)	13 (5–30)	12 (6–31)	14 (4–30)		0.93^b^
Subarachnoid extension, n (%)	25 (19)	11 (29)	14 (18)	1.0 (0.7–1.5)^c^	
Intraventricular extension, n (%)	49 (36)	22 (39)	27 (35)	1.1 (0.8–1.5)^c^	
ICH subtype, n (%)					
CAA	39 (29)	16 (28)	23 (30)		0.85^c^
Arteriolosclerosis	45 (33)	20 (35)	25 (32)		
Mixed small vessel disease	50 (37)	21 (37)	39 (37)		
Not possible	1 (1)	0 (0)	1 (1)		

Abbreviations: BP = blood pressure; CMI = cerebral microinfarct; GCS = Glasgow Coma Scale; IQR = interquartile range; NIHSS = NIH Stroke Scale; SAH = subarachnoid hemorrhage; sICH = spontaneous intracerebral hemorrhage.

a *t* test.

b Mann-Whitney *U* test.

c Chi-squared test or Fisher exact test.

On 3T MRIs, we found a total of 100 CMIs in 57 of 135 patients (42%). The CMIs were identified in 41% of 58 patients with lobar sICH and 43% of 77 patients with non-lobar sICH. The number of CMIs per patient ranged from 1 to 4; 29 patients had more than 1 CMI (median 2, IQR 1–2). Of the 100 CMIs, 52 were found in the frontal cortex, 22 parietal, 15 temporal, and 12 occipital. The spatial distribution was comparable between lobar and non-lobar sICH patients. None of the CMIs corresponded to DWI lesions on 3T MRI, so all CMIs were presumed to be chronic.

Patients with CMIs were older (mean [SD] 66 [13] years, for patients with CMIs [CMI+] vs 61 [15] years, for patients without CMIs [CMI−], *p* = 0.04). A history of TIA or ischemic stroke was associated with presence of CMIs (CMI+ 23% vs CMI− 5%; risk ratio [RR] 2.7, 95% CI 1.1–6.4). In patients with CMIs, the systolic blood pressure at presentation was comparable with those without CMIs (171 mm Hg [SD 30] vs 168 mm Hg [SD 34], *p* = 0.65). Sex, other vascular risk factors, and hematoma characteristics were comparable in patients with and without CMIs. Presence of CMIs was not associated with probable CAA according to the Boston criteria version 2.0 or ICH subtype according to CLAS-ICH (Table 1).

In patients with CMIs, we found a higher median volume of WMH (CMI+ 8.6 mL (IQR 6.7–19.6) vs CMI 3.7 mL (IQR 1.5–11.9); *p* = 0.001). We found the same association when WMH volume was expressed as percentage of ICV and when WMH were rated with the Fazekas score and the association was comparable between lobar and non-lobar sICH patients. CMIs were not associated with CMBs, DWI lesions, PVS, cSS, or lacunes (Table 2).

**Table 2:** 3T MRI Markers for Patients With Lobar and Non-Lobar sICH With and Without Presence of Cortical CMIs on 3T MRI

Characteristics	All (N = 135)	CMI+ (N = 57, 42%)	CMI− (N = 78, 58%)	Risk ratio (95% CI)	*p* Value
CMBs^a^					
Presence, n (%)	75 (56)	35 (65)	40 (51)	1.2 (0.9–1.6)^f^	
Categories					
0	53 (39)	19 (33)	34 (44)		Ref^g^
1–10	52 (39)	27 (47)	25 (32)		0.10
11–50	20 (15)	7 (12)	13 (17)		0.95
>50	3 (2)	1 (2)	2 (3)		0.93
Any lobar CMBs, n (%)	58 (43)	25 (44)	33 (42)	1.0 (0.8–1.4)^f^	
Strictly lobar CMBs, n (%)	17 (13)	10 (18)	7 (9)	1.5 (0.8–2.6)^f^	
Number of lobar CMBs, median (IQR)	0 (0–3)	0 (0–3)	0 (0–3)		0.99^h^
Number of non-lobar CMBs, median (IQR)	0 (0–3)	0 (0–3)	0 (0–2)		0.95^h^
Total number of CMBs, median (IQR)	1 (0–5)	1 (0–5)	1 (0–6)		0.55^h^
WMHs^b^					
WMH volume, mL, median (IQR)	5.7 (2.1–14.1)	8.6 (4.6–18.3)	3.7 (1.5–11.9)		0.001^h^
WMH volume, % of ICV volume, median (IQR)	0.4 (0.2–1.0)	0.5 (0.3–1.2)	0.3 (0.1–0.9)		0.002^h^
Periventricular WMH Fazekas score, median (IQR)	1.5 (1–2)	2 (1–3)	1 (1–2)		0.03^h^
Deep WMH Fazekas score, median (IQR)	2 (1–2)	2 (1–2)	1 (1–2)		0.004^h^
Lobar ICH (n = 58)					
WMH volume, mL, median (IQR)	7.6 (2.9–18.6)	11.3 (6.7–19.6)	5.2 (2.3–17.8)		0.047^h^
WMH volume, % of ICV volume, median (IQR)	0.5 (0.2–1.3)	0.7 (0.5–1.5)	0.4 (0.2–1.2)		0.051^h^
Non-lobar ICH (n = 77)					
WMH volume, mL, median (IQR)	4.8 (1.5–12.5)	7.9 (3.2–14.9)	3.2 (1.4–8.8)		0.013^h^
WMH volume, % of ICV volume, median (IQR)	0.3 (0.1–0.8)	0.5 (0.2–1.0)	0.2 (0.1–0.6)		0.018^h^
DWI lesions^c^					
Presence, n (%)	24 (18)	9 (16)	15 (19)	0.9 (0.6–1.3)^f^	
Number of DWI lesions, median (IQR)	0 (0–0)	0 (0–0)	0 (0–0)		0.56^h^
Perivascular spaces^d^					
Severe number of PVS (>20) in basal ganglia, n (%)	47 (35)	21 (37)	26 (33)	1.0 (0.8–1.4)^f^	
Severe number of PVS (>20) centrum semiovale, n (%)	89 (66)	41 (72)	48 (62)	1.2 (0.9–1.6)^f^	
Cortical superficial siderosis^a^					
Presence, n (%)	22 (16)	11 (19)	11 (14)	1.2 (0.8–1.8)^f^	
Focal	12 (9)	5 (9)	7 (9)		0.52^f^
Disseminated	10 (7)	6 (11)	4 (5)		
Lacunes^e^					
Presence, n (%)	23 (17)	12 (21)	11 (14)	1.3 (0.8–2.0)^f^	
Number of lacunes, median (IQR)	0 (0–0)	0 (0–0)	0 (0–0)		0.23^h^
Boston 2.0 criteria					
Probable CAA, n (%)	32 (24)	12 (21)	20 (26)	0.9 (0.7–1.2)^f^	
Possible CAA, n (%)	7 (5)	5 (9)	3 (4)	1.6 (0.6–3.9)^f^	

Abbreviations: CAA = cerebral amyloid angiopathy; CMB = cerebral microbleed; CMI = cerebral microinfarct; DWI = diffusion-weighted imaging; ICV = intracranial brain volume; IQR = interquartile range; MD = mean diffusivity; PVS = perivascular spaces; sICH = spontaneous intracerebral hemorrhage; WMH = white matter hyperintensity.

a Eleven scans were not assessable due to insufficient quality.

b Nine scans were not assessable due to insufficient quality.

c Fifteen scans were not assessable due to insufficient quality.

d One scan was not assessable due to insufficient quality.

e Six scans were not assessable due to insufficient quality.

f Chi-squared test or Fisher exact test.

g Logistic regression.

h Mann-Whitney *U* test.

In a multivariable model with age, sex, history of TIA or ischemic stroke, and median WMH volume, only history of TIA and ischemic stroke remained associated with presence of CMIs (adjusted odds ratio [aOR] 4.7, 95% CI 1.4–16.6).

### 7T MRI

Of the 204 included patients in the FETCH study, 45 underwent a 7T MRI. In 5 patients, the quality of the FLAIR or T_1_-weighted images was insufficient due to movement artefacts, and therefore, 40 patients were available for analysis. Characteristics of the 40 included patients are listed in Table 3. The median time interval between sICH and 7T MRI was 36 days (IQR 9–71 days). Median hematoma volume was 11 mL (IQR 4–19 mL). Location of the sICH was lobar in 12 patients (30%) and non-lobar in 28 patients (70%; 22 deep, 6 infratentorial).

**Table 3:** Baseline Characteristics and MRI Markers for Patients With Lobar and Non-Lobar sICH With and Without Presence of Cortical CMIs on 7T MRI

Characteristics	All (N = 40)	CMI+ (N = 28, 70%)	CMI− (N = 12, 30%)	Risk ratio (95% CI)	*p* Value
Patient characteristics					
Age, y, mean (SD)	59 (13)	62 (12)	52 (14)		0.04^d^
Female sex, n (%)	12 (30)	10 (35)	2 (17)	2.1 (0.6–8.3)^f^	
GCS at presentation, median (IQR)	15 (12–15)	15 (13–15)	14 (12–15)		0.46^e^
NIHSS at presentation, median (IQR)	4 (2–9)	4 (2–10)	4 (1–6)		0.42^e^
Systolic BP at admission, mm Hg, mean (SD)	171 (33)	177 (31)	156 (33)		0.07^d^
Diastolic BP at admission, mm Hg, mean (SD)	96 (22)	97 (21)	94 (23)		0.65^d^
History of hypertension, n (%)	24 (60)	19 (68)	5 (42)	2.1 (0.8–5.5)^f^	
History of TIA or ischemic stroke, n (%)	8 (20)	7 (25)	1 (8)	2.8 (0.4–18.3)^f^	
History of intracerebral hemorrhage, n (%)	2 (5)	2 (7)	0 (0)		
History of subarachnoid hemorrhage, n (%)	0 (0)	0 (0)	0 (0)		
History of cardiac disease, n (%)	2 (5)	1 (4)	1 (8)	0.6 (0.1–2.5)^f^	
History of peripheral artery disease, n (%)	2 (5)	2 (7)	0 (0)		
Diabetes mellitus, n (%)	2 (5)	2 (7)	0 (0)	1.2 (0.7–2.1)^f^	
Hypercholesterolemia, n (%)	11 (28)	8 (29)	3 (25)		
Atrial fibrillation, n (%)	5 (13)	4 (14)	1 (8)	1.6 (0.3–9.7)^f^	
Smoking (current or past), n (%)	18 (45)	13 (45)	5 (42)	1.2 (0.5–3.1)^f^	
Alcohol use, n (%)^a^	31 (78)	21 (75)	10 (83)	1.1 (0.6–1.8)^f^	
Alcohol use ≥15 units/week, n (%)^a^	4 (10)	2 (7)	2 (17)	1.4 (0.5–3.9)^f^	
Anticoagulants, n (%)	7 (18)	6 (21)	1 (9)	2.3 (0.4–15.3)^f^	
Antiplatelets, n (%)	8 (20)	6 (21)	2 (17)	1.3 (0.3–4.6)^f^	
Hematoma characteristics					
Location, n (%)					
Lobar	12 (30)	8 (28)	4 (36)		0.59^f^
Deep	22 (55)	17 (59)	5 (46)		
Infratentorial	6 (15)	4 (14)	2 (18)		
Hematoma volume, mL, median (IQR)	11 (4–19)	11 (4–15)	19 (3–28)		0.31^e^
Subarachnoid extension, n (%)	6 (15)	2 (7)	4 (33)	0.4 (0.1–1.4)^f^	
Intraventricular extension, n (%)	11 (28)	5 (18)	6 (50)	0.6 (0.3–1.1)^f^	
MRI markers					
Presence of CMBs^b^					
CMBs (yes, %)	29 (73)	24 (86)	5 (42)	3.5 (1.4–8.9)^f^	
Any lobar CMBs (yes, %)	22 (55)	18 (64)	4 (33)	2.3 (0.8–6.5)^f^	
Strictly lobar CMBs (yes, %)	5 (13)	4 (14)	1 (8)	1.5 (0.2–9.2)^f^	
Number of CMBs					
Number of lobar CMBs, median (IQR)	1 (0–6)	2 (0–8)	0 (0–2)		0.12^e^
Number of non-lobar CMBs, median (IQR)	1 (0–5)	2 (0–8)	0 (0–1)		0.13^e^
Total number of CMBs, median (IQR)	3 (0–8)	3 (1–10)	0 (0–3)		0.03^e^
Contrast leakage^c^					
Presence, n (%)	16 (40)	13 (46)	3 (25)	2.0 (0.6–6.4)^f^	
HARM scale score ≥3, n (%)	7 (18)	4 (14)	3 (25)	0.6 (0.2–1.7)^f^	

Abbreviations: BP = blood pressure; CMB = cerebral microbleed; CMI = cerebral microinfarct; FLAIR = fluid-attenuated inversion recovery; GCS = Glasgow Coma Scale; HARM = hyperacute injury marker; IQR = interquartile range; NIHSS = NIH Stroke Scale; sICH = spontaneous intracerebral hemorrhage.

a Information not available in 2 patients.

b In 1 patient, CMBs were not assessable due to movement artifacts.

c In 5 patients, FLAIR images after gadolinium were not available or assessable.

d *t* test.

e Mann-Whitney *U* test.

f Chi-squared test.

We found a total of 59 CMIs in 28 of 40 (70%) patients (in 8/12 lobar [67%] and 20/28 non-lobar sICH patients [71%], RR 0.9, 95% CI 0.6–1.5). The number of CMIs per patient ranged from 1 to 10; 14 patients had more than 1 CMI (median 2, IQR 1–3). Of the 59 CMIs, 28 (47%) were found in the frontal cortex, 18 parietal (31%), 5 temporal (8%), and 8 occipital (14%). The spatial distribution was comparable between lobar and non-lobar sICH patients. Illustrative examples are provided in Figure. Patients with CMIs were older (CMI+ mean 62 [12] years, for vs CMI− 52 [14] years, *p* = 0.04). In patients with CMIs, the systolic blood pressure at presentation was comparable with those without CMIs (177 mm Hg [SD 31] vs 156 mm Hg [SD 33], *p* = 0.07). On admission CT, we found subarachnoid extension in 7% of patients with CMIs compared with 33% in patients without CMIs (*p* = 0.06). Sex, vascular risk factors, and other hematoma characteristics were comparable in patients with and without CMIs on 7T MRI.

**Figure:**
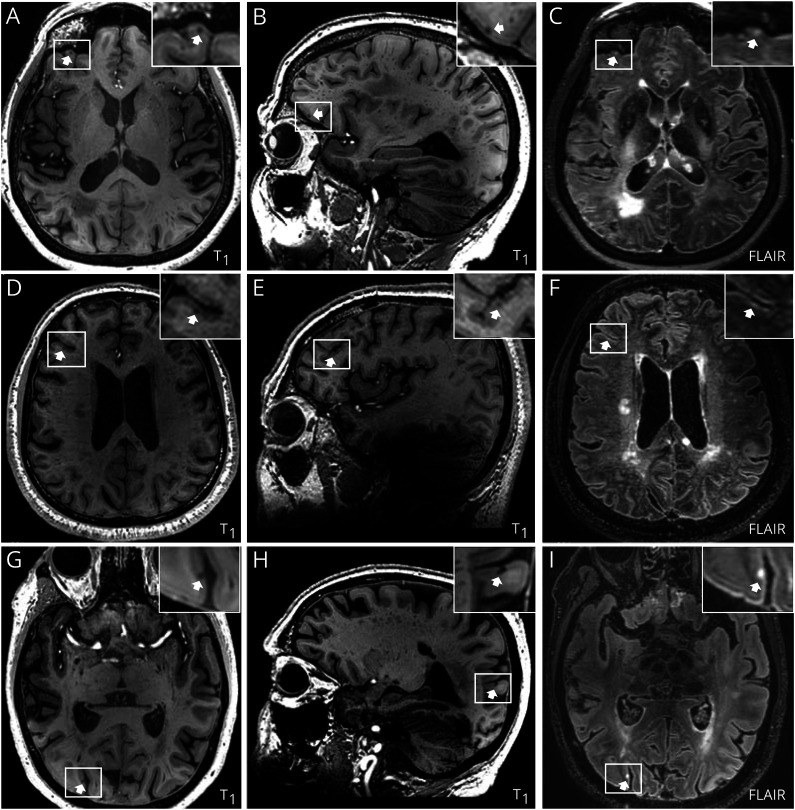
Examples of CMIs in Patients With sICH Panels A–C show the 7T MRIs of a patient with a right lobar sICH, fulfilling the Boston criteria version 2.0 of probable cerebral amyloid angiopathy, and a CMI (white arrows) in the superficial part of the cortex (A: axial T_1_-weighted image; B: sagittal T_1_-weighted image; C: axial FLAIR image). Panels D–F depict a patient with a left deep sICH and a CMI (white arrows) in the deeper part of the cortex (D: axial T_1_-weighted image; E: sagittal T_1_-weighted image; F: axial FLAIR image). Panels G–I show images of a patient with a right deep sICH and a transcortical CMI (white arrows; G: axial T_1_-weighted image; H: sagittal T_1_-weighted image; I: axial FLAIR image). The inserts show the CMIs in more detail. CMI = cerebral microinfarct; FLAIR = fluid-attenuated inversion recovery; sICH = spontaneous intracerebral hemorrhage.

Of the 40 patients with a 7T MRI, 35 patients also had an assessable 3T MRI. In 10 of the 28 patients with 1 or more CMI on 7T MRI (median 2, IQR 1–3), we also found 1 or more CMI on the 3T MRI (median 2, IQR 1–3). In 5 patients, we found more CMIs on 7T MRI and in 3 patients more CMIs on 3T MRI.

Patients with CMIs more often had any CMBs (86%) than those without CMIs (42%; RR 3.5, 95% CI 1.4–8.9) and a higher total number of CMBs (CMI+ median 3, IQR 1–10 vs CMI− median 0, IQR 0–3; *p* = 0.03). In patients with CMIs, we found a median WMH volume of 10 mL (IQR 4–13) compared with 2 mL (IQR 1–5) in patients without CMIs (*p* = 0.054). CMIs were not associated with contrast leakage (Table 3).

In multivariable models correcting for age and sex, CMIs on 7T MRI were no longer associated with presence (aOR 4.8, 95% CI 0.9–26.8) or total number (aOR 1.05, 95% CI 0.95–1.16) of CMBs.

Of the 59 CMIs, 8 were located in the superficial layers of the cortex, 22 in the deeper layers of the cortex, and 29 were considered transcortical. The location of the CMIs in the cortex was more often only superficial in patients with lobar sICH (30%) compared with patients with non-lobar sICH (5%; RR 2.7, 95% CI 1.5–5.0; Table 4 and Figure). The percentage of CMIs with deep (*p* = 0.29) or transcortical (*p* = 0.06) location in the cortex was similar in lobar and non-lobar sICH patients. We explored the location of the CMIs in the cortex in the ICH subtypes according to CLAS-ICH etiology and found no significant differences (Table 5).

**Table 4:** Location of CMIs in the Cortex on 7T MRI in Patients With Lobar and Non-Lobar sICH

Location of CMIs in the cortex	Lobar sICH (8 patients; 20 CMIs)	Non-lobar sICH (20 patients; 39 CMIs)	Risk ratio (95% CI)^a^
Superficial, n (%)	6 (30)	2 (5)	2.7 (1.5–5.0)
Deep, n (%)	6 (30)	16 (41)	0.8 (0.6–1.2)
Transcortical, n (%)	8 (40)	21 (54)	0.9 (0.6–1.2)

Abbreviations: CMI = cerebral microinfarct; sICH = spontaneous intracerebral hemorrhage.

a Chi-squared test.

**Table 5:** Location of CMIs in the Cortex on 7T MRI Based on Intracerebral Hemorrhage Subtype According to CLAS-ICH

Location of CMIs in the cortex	Arteriolosclerosis (11 patients; 19 CMIs)^a^	CAA (5 patients; 16 CMIs)^b^	Mixed SVD (12 patients; 24 CMIs)^c^	Risk ratio (95% CI)^d^
Superficial, n (%)	2 (10)	2 (13)	4 (17)	1.3 (0.4–4.7)
Deep, n (%)	7 (37)	6 (37)	9 (38)	0.9 (0.4–1.9)
Transcortical, n (%)	10 (53)	8 (50)	11 (45)	1.0 (0.5–2.1)

Abbreviations: CAA = cerebral amyloid angiopathy; CMI = cerebral microinfarct; SVD = small vessel disease.

a One patient 4 CMIs, 1 patient 3 CMIs, 3 patients 2 CMIs, and 6 patients 1 CMI.

b One patient 10 CMIs, 2 patients 2 CMIs, and 2 patients 1 CMI.

c One patient 7 CMIs, 1 patient 3 CMIs, 4 patients 2 CMIs, and 6 patients 1 CMI.

d Chi-squared test; arteriolosclerosis vs CAA and mixed SVD.

## Discussion

In our cohort of sICH patients, we found CMIs in less than half of patients on 3T MRI and in 70% of patients on 7T MRI, irrespective of hematoma (i.e., lobar or non-lobar) location. The presence of CMIs on 3T MRI was related to history of TIA or ischemic stroke, but not associated with other vascular risk factors or MRI markers of cSVD. The presence of CMIs on 7T MRI was not related to vascular risk factors and other MRI markers of cSVD. In lobar sICH compared with non-lobar sICH patients, CMIs were more often exclusively located in the superficial part of the cortex on 7T MRI.

This study provides evidence that CMIs are a common finding in patients with sICH. CMIs are not uncommon in other types of strokes and vascular disease with proportions varying from 15% in patients with TIA or ischemic stroke to 30% in patients with cardiac disease.^29,30^ CMIs are also often found in patients with CAA with and without a lobar sICH with percentages up to 60%.^31-33^ Based on our observations and those of others, the presence of CMIs does not seem to be specific for sICH, or for stroke in general, but CMIs seem to be related to older age and cardiovascular disease.

The etiology of CMIs is unclear. They are a possible manifestation of cSVD, but also large vessel atherosclerosis plays a role.^34^ Other suggested mechanisms are hypoperfusion, blood pressure variability, and microemboli.^35,36^

In a small case series of 12 patients and 15 controls, we previously found that on 7T MRI, CMIs are observed in most sICH patients.^15^ This study extends those preliminary findings and suggests that cortical CMIs occur both in patients with lobar and non-lobar sICH.^15^

We found no clear associations between CMIs and other MR markers of cSVD. On 3T MRI, we found that presence of CMIs was related to severity (scored with the Fazekas scale) and median volume of WMH in univariable analysis. This is in line with 2 population-based studies and a memory clinic cohort that showed a relation of CMIs assessed on 3T MRI with higher WMH volume.^37-39^ In a study of 102 patients with CAA, an association was found with cSS.^31^ We could not confirm this relation in the patients with CAA in our cohort, but in our study, the number of patients with probable CAA was small (n = 32 with 3T MRI and n = 6 with 7T MRI) and the proportion of CAA patients with cSS was even smaller. We found a possible connection of occurrence of CMIs with CMBs on 7T MRI. We found the link for presence and number of CMBs irrespective of their location, and not specifically for strictly lobar CMBs. This is in line with a meta-analysis that showed a relation between prevalence of acute CMIs visualized with DWI and presence and number of CMBs in any location.^40^ In the studies of CAA patients with or without previous lobar ICH, no associations were found between presence of CMIs and presence or number of lobar CMBs.^31,32^ Another study found a link between presence of CMIs on pathology and MRI-visible CMBs.^41^ Our findings show that CMIs are found in both lobar and non-lobar sICH with CMBs in any location. This suggests that CMIs are not specific to CAA, but also related to other types of cSVD-related ICH.

We found that in patients with sICH, a history of TIA or ischemic stroke was related to presence of CMIs on 3T MRI, but not to other vascular risk factors. A previous 3T MRI study in memory clinic patients also found a relation with a history of ischemic stroke and furthermore a link with a history of cardiac disease and hyperlipidemia.^37^ Because no relationship was found between presence of CMIs and older age, they did not adjust for age in that study.^37^ In the 3T MRI cohort of patients with TIA and ischemic stroke, no association was found with older age and also none of the investigated risk factors showed a link with presence of CMIs.^29^ In a population-based study with 3T MRI, 6% had 1 or more CMIs and presence of CMIs was related to hypertension and older age.^39^ A previous 7T MRI study did not observe a link between presence and number of CMIs and type 2 diabetes mellitus.^42^ It seems that the disease setting is important in interpreting associations of vascular risk factors with CMIs.^22^ Owing to our selection of patients with sICH, risk factor patterns were different than in the general population or a memory clinic setting. It remains unclear what the influence is of vascular risk factors on the development of CMIs, but older age is probably an important modifying factor.

In line with our postmortem study combining 7T MRI and histopathology,^16^ we found CMIs more often in the superficial part of the cortex in lobar sICH patients compared with non-lobar sICH patients on 7T MRI. This is consistent with a study using ex vivo 3T MRI and histopathology in 12 patients with CAA, which showed that half of the CMIs were located in the superficial layers of the cortex, in the perfusion area of a penetrating cortical arteriole.^43^ Another neuropathologic study, including 113 consecutive brain autopsies, showed that CAA predominantly affected the superficial cortical branches of pial arteries.^44^ Combined with our study, these findings suggest that CAA might specifically damage the superficial branches of cortical arteries with superficial cortical CMIs as a consequence and that arteriolosclerotic vasculopathy specifically damages the deeper branches of cortical arteries with deep cortical CMIs as a result.

Strengths of our study include the unique cohort of sICH patients who underwent 3T and ultra-high field 7T MRI. The high signal-to-noise ratio of 7T MRI allows detection of smaller lesions, and therefore, more CMIs are found than on conventional MRI.^45^ Indeed, we found CMIs in more patients who underwent 7T MRI (70%), than in patients who had 3T MRI (42%). In addition, we were able to scan all patients in the (sub)acute phase after the sICH and around half of the patients within a month.

Our study also has limitations. Selection bias has likely played a role. It is challenging to include severely disabled sICH patients in an MRI study. This may have resulted in including less affected and relatively young patients with lower NIHSS scores and possibly smaller hematomas. Information bias might have been introduced because we were not able to perform blinded assessments of MRI markers for sICH location. Another limitation is that although this series is unique in numbers of patients with sICH undergoing 7T MR early after the hemorrhage, still the number of patients may have been too small to find possible associations with modest effect sizes with vascular risk factors and MRI markers.

CMIs are a common finding in patients with lobar and with non-lobar sICH. Patients with lobar sICH, more often have CMIs in the superficial part of the cortex than those with non-lobar sICH. Location of CMIs within the cortex might therefore provide insight into the underlying type of small vessel disease in sICH, being either arteriolosclerosis or CAA. To further elucidate the meaning of presence of CMIs in sICH, it would be of interest to investigate associations of presence and number of CMIs with functional outcome, with recurrent stroke and with cognitive function and decline, both in patients with lobar and non-lobar sICH. Moreover, serial imaging could be used to investigate the role of CMIs in progression of cognitive decline and to determine their importance for choices regarding antithrombotic treatment in patients with sICH.

## Glossary

ADC: apparent diffusion coefficient
aOR: adjusted odds ratio
CAA: cerebral amyloid angiopathy
CLAS-ICH: Causal Classification System for Intracerebral Hemorrhage Subtypes
CMB: cerebral microbleed
CMI: cerebral microinfarct
cSS: cortical superficial siderosis
cSVD: cerebral small vessel disease
DWI: diffusion-weighted image
FETCH: Finding the ETiology in Spontaneous Cerebral Hemorrhage
FLAIR: fluid-attenuated inversion recovery
HARM: hyperacute injury marker
ICV: intracranial volume
IQR: interquartile range
NIHSS: NIH Stroke Scale
PVS: perivascular space
sICH: spontaneous intracerebral hemorrhage
SWI: susceptibility weighted image
WMH: white matter hyperintensity
